# Characterization of microRNA Profiles in *Pasteurella multocida*-Infected Rabbits and Identification of miR-29-5p as a Regulator of Antibacterial Immune Response

**DOI:** 10.3389/fvets.2021.746638

**Published:** 2021-11-17

**Authors:** Jiaqing Hu, Wenqiang Li, Xibo Qiao, Wenjie Li, Kerui Xie, Yanyan Wang, Bing Huang, Qiaoya Zhao, Lei Liu, Xinzhong Fan

**Affiliations:** ^1^Shandong Provincial Key Laboratory of Animal Biotechnology and Disease Control and Prevention, College of Animal Science and Veterinary Medicine, Shandong Agricultural University, Taian, China; ^2^Shandong New Hexin Technology Co. Ltd., Taian, China; ^3^Shandong Provincial Key Laboratory of Poultry Disease Diagnose and Immune, Institute of Poultry, Shandong Academy of Agricultural Sciences, Jinan, China

**Keywords:** *Pasteurella multocida*, small RNA-seq, ocu-miR-29-5p, target gene, antibacterial immune

## Abstract

*Pasteurella multocida* is the pathogenic agent for a variety of severe diseases in livestock, including rabbits. MicroRNAs (miRNAs) participate in the immune response to the pathogen. Distinct miRNA expression patterns were explored in rabbit lung by small-RNA deep sequencing to assess dysregulated miRNAs during *P. multocida* infection. Totally, 571 miRNAs were screened, of which, 62 were novel, and 32 exhibited differential expression (DE). Of the 32 known DE-miRNAs, 13 and 15 occurred at 1 day and 3 days post-infection (dpi); and ocu-miR-107-3p and ocu-miR-29b-5p were shared between the two time points. Moreover, 7,345 non-redundant target genes were predicted for the 32 DE-miRNAs. Putative target genes were enriched in diverse GO and KEGG pathways and might be crucial for disease resistance. Interestingly, upregulation of ocu-miR-29-5p suppresses *P. multocida* propagation and downregulates expression of epithelial membrane protein-2 (EMP2) and T-box 4 (TBX4) genes by binding to their 3′ untranslated region in RK13 cells. Thus, ocu-miR-29-5p may indirectly inhibit *P. multocida* invasion by modulating genes related to the host immune response, such as EMP2 and TBX4.

## Introduction

*Pasteurella multocida* (*P. multocida*) is a pathogenic Gram-negative bacterium that frequently infects the respiratory tract of most livestock and causes significant economic loss worldwide (1, 2). *P. multocida* strains/isolates are grouped into five serogroups—A, B, D, E, and F—based on capsule antigens. Further, 16 serotypes are recognized using lipopolysaccharide (LPS) antigens (3, 4). Rabbits can become infected with *P. multocida* immediately after birth. The infection causes rhinitis in the upper respiratory and pneumonia in the lower respiratory tracts (1, 5). Notably, prevalence rates as high as 94% are reported for *P. multocida*, and, the majority of adult rabbits are recognized as carriers (6). Besides, *P. multocida* can also cause human infections via animal bites and/or scratches (2, 7, 8).

Small noncoding ribonucleic acid (snRNA)—microRNA (miRNA), siRNA, and piRNA—have been purified from many mammals (9). Among the snRNA, most studies have focused on miRNA, which are recently reported endogenous non-coding RNA molecules (a length of approximately 19–25 nucleotides) that are highly conserved across species (10). They combine with target mRNAs and down-regulate or degrade the mRNA (11). This action initiates the innate immune response through secretion of cytokines/chemokines as well as participated in multiple signaling pathways and biological processes, such as embryonic development, proliferation, differentiation, energy metabolism, and inflammation (12–15).

Animals rapidly alter gene expression patterns in response to pathogens and miRNA are key modulatory biomolecules for such responses (16). However, pathogens counter host defenses, particularly through targeting regulation mediated by miRNAs (17). For example, miR-146 expression is elevated in response to many pathogens including *Helicobacter pylori* (18), *Listeria monocytogenes* (19), and *S. Typhimurium* (20). This factor is involved in the regulation of essentially innate immune responses, including biosynthesis of proinflammatory cytokine TNF-α and IL-1β and modulation of the migration of immune cell (21). Moreover, miR-125b is a crucial modulator of host inflammatory responses through the TLR cascade and directly targets TNF-α to clear the pathogens (22, 23).

In contrast, the function of miRNAs in rabbit immunity during *P. multocida* infection has not yet been reported. Thus, we focused on miRNA expression profiles using RNA-seq in rabbit lungs after infection with *P. multocida* and examined the predicted target genes of *P. multocida*-induced differential expression (DE)-miRNAs. Additionally, we identified the role of ocu-miR-29-5p in conferring antibacterial immunity to *P. multocida* infection.

## Materials and Methods

### Ethics Statement, Pathogen Challenging and Sample Collection

All animal studies were conducted according to the guidelines of the Institutional Animal Care and Use Committee of Shandong Agricultural University (Approval Number: #SDAUA-2018-028) and the “Guidelines for Experimental Animals” of the Ministry of Science and Technology (Beijing, China). New Zealand rabbits, 38-days-old, were obtained from Qingdao Kangda Rabbit Industry Development Co., Ltd. All nasopharyngeal cultures of these animals tested negative for *P. multocida*. Moreover, rabbit sera were also negative for *P. multocida* based on ELISA using a specific antibody against the bacterium. Rabbits were adapted to their environment for one week before use in experiments.

Twenty-five rabbits were randomized into test (*n* = 20) and control (*n* = 5) groups. For the challenge assay, rabbits were injected subcutaneously with 10^7^ CFU of strain type A3 (LD 50 = 2.3 × 10^7.8^ CFU/mL) in 1mL PBS (24). One day post-infection (P1, dpi) and 3 dpi (P3), five rabbits with symptoms of depression, anorexia, snuffles, serous nasal exudate, and dyspnea were euthanized by pentobarbital overdose (100 mg/kg, intravenous). Unchallenged rabbits (P0) were used as a negative control. All the lung samples were collected under aseptic conditions and immediately stored at −80°C. The remining alive rabbits, post-experiment, were euthanized by the same procedure.

### miRNA Library Construction, Sequencing and Identification

Every three samples we used for the RNA-seq were taken at P0, P1, and P3 to examine the lung tissues at different stages of infection. Total RNA was extracted from lung tissues using a TRIzol Kit (Invitrogen, CA, USA), as recommended by standard procedure. RNA purity and integrity were checked through NanoDrop 2000 Spectrophotometer and Agilent 2100 Bioanalyzer. MiRNA enrichment was done with the PureLink miRNA isolation Kit (Invitrogen, USA) for library construction. Briefly, 15% urea denaturing polyacrylamide gel electrophoresis (PAGE) was employed to purify the miRNAs molecules (<30 nucleotides). Next, miRNAs were ligated with the specified adaptors at the 3′ and 5′ ends. Subsequently, ligated miRNAs were converted into cDNA, and products were successively amplified with PCR. A second process of size selection was performed, and target products were excised from gels. Finally, products were purified and submitted for miRNA transcriptome sequencing (Huada Gene, Shenzhen, Guangdong, China).

Adaptor sequences, redundant contaminants, low-quality reads were eliminated to ensure reliable clean data. Clean reads after data filtering were mapped with known small-RNA data resources—miRbase, snoRNA, Rfam, piRNA, and siRNA—to remove additional non-coding RNAs (rRNA, tRNA, snoRNA, and snRNA) and repeat sequences (25, 26). miRDeep2 (27) was used to predict novel miRNA by exploring secondary structure, and Piano (28) was used to predict piRNAs. miRNA expression level was calculated by Fragments per Kilobase Million (FPKM) method (29). Differential expression (DE) analysis used DEGseq (30) with a threshold of Q value ≤ 0.001 and |Log2 (fold changes)| ≥ 1.

### miRNA Target Genes Prediction and Annotation

To predict genes targeted by DE-miRNAs, putative 3′-UTR of rabbit mRNA were used with two computational algorithms RNAhybrid (31) and miRanda (32). Moreover, explicit roles of miRNA-targeted genes in distinct biological processes were explored with the Gene Ontology (GO) and Kyoto Encyclopedia of Genes and Genomes (KEGG) databases. Notable terms and pathways were adjusted using a Q value with a rigorous Bonferroni cutoff (33).

### Real-Time Quantitative PCR (RT-qPCR) Assay

DE-miRNAs identified by sequencing were selected for the validation of expression results. mRNA abundance for target genes was also examined. An miRcute miRNA isolation kit (Tiangen, Beijing, China) was employed to isolate miRNAs, and Trizol reagent (Invitrogen, CA, USA) was performed to extract total RNA from rabbit lung samples following manufacturer instructions. RNA was processed with the miRcute Plus miRNA First-Strand cDNA Synthesis Kit (Tiangen, Beijing, China) and PrimeScript™ RT reagent kit with gDNA Eraser (Takara, Dalian, China) on the basis of manufacturer recommended. DE-miRNA expression level was validated with an miRcute Plus miRNA qPCR Kit (SYBR Green, Tiangen), and U6 was quantified as the internal control gene. mRNA expression profiles were quantified with RT-qPCR, normalized to the level of GAPDH mRNA (34). Each sample was run in triplicate, and relative miRNA target gene expression was calculated using the 2^−ΔΔCT^ approach (35). The primer sequences were listed in Table 1.

**Table 1:** Primers sequences for the RT-qPCR confirmation of the selected miRNA and their target gene.

**miRNA/mRNA**	**Primers (5′-3′)**
ocu-miR-212-3p	CCTAACAGTCTCCAGTCACGGC
ocu-miR-155-3p	CGCCGCTCCTACATGTTAGCATTAAC
ocu-miR-128a-3p	CGTCACAGTGAACCGGTCTCTTT
ocu-miR-21-3p	CAACAGCAGTCGATGGGCTGT
ocu-miR-122-3p	GCGCGAACGCCATTATCACACTAAATA
ocu-miR-148b-5p	GCCGAAGTTCTGTTATACACTCAGGCT
ocu-miR-215-5p	CGCCGTTGACCTATGAAATGACAGATG
ocu-miR-206-3p	CCGTGGAATGTAAGGAAGTGTGTGG
ocu-miR-29b-5p	GCGCTGGTTTCATATGGTGGTTTAGA
U6-F	CTCGCTTCGGCAGCACA
U6-R	AACGCTTCACGAATTTGCGT
GAPDH-F	TCACAATCTTCCAGGAGCGA
GAPDH-R	CACAATGCCGAAGTGGTCGT
EMP2-F	TGGTGGGTCGGAGAGGAGTTTG
EMP2-R	ACAGCAGAGGATGGTGGACAGG
TBX4-F	GCAGCACTACCAGTACGAGAACG
TBX4-R	TGGGCAGGGAAGGTATTGAGAGG

### Transfection and Cellular Infection

Rabbit kidney (RK13) cells were grown in DMEM medium (Gibco, USA) containing 10% heat-inactivated fetal bovine serum (FBS, Gibco, USA) and 1% antibiotics. Transient transfections of RK13 cells with the ocu-miR-29-5p mimic or a negative control mimic or inhibitor or negative control inhibitor used lipofectamine 2000 (Invitrogen), and all procedures strictly followed standard instructions. After optimization, a final oligonucleotide concentration of 30 nM per well was adopted. Knockdown of ocu-miR-29-5p expression used an inhibitor or control inhibitor at a final oligonucleotide concentration of 80 nM. The transfection medium was incubated for 6 h, then replaced with complete culture medium and incubated for another 24 h.

RK13 cells were challenged with *P. multocida* at the multiplicity of infection (MOI) of 10 and harvested at different times (0, 6, 12, and 24 h) for RNA extraction. Uninfected RK13 cells served as negative control, and each trial contained three biological replicates. To determine the effect of ocu-miR-29-5p and inhibitor on *P. multocida* replication, the RK13 cells (2 × 10^5^ cells/well) were pre-transfected with 30 nM ocu-miR-29-5p mimic or negative control mimic or inhibitor or negative control inhibitor. Three hours after challenge, cells were harvested and washed three times with PBS containing gentamicin (100 ug/mL) for 3 h to kill extracellular *P. multocida*. Cells were then cultured for 3 h in DMEM containing gentamicin and the cells were lysed using 1% Triton X-100 in PBS for 20 min. Intracellular bacterial loads (CFU/mL) were assessed via plating of lysates onto nutrient agar.

### Luciferase Reporter Assay

To further confirm the interactions between miR-122 and target genes, the 3′-untranslated region (3′ UTR) sequence of epithelial membrane protein-2 (EMP2) and T-box gene 4 (TBX4) was cloned into the XhoI/NotI restriction sites of the psiCheck2 vector. Recombinant plasmids were verified by DNA sequencing and re-named EMP2 3′ UTR WT and TBX4 3′ UTR WT, respectively. The EMP2 and TBX4 3′ UTR sequence complementary to the ocu-miR-29-5p seed sequence (UUUGGUC) was then mutated to GGGAAGA, and the reconstructed mutated clones were re-named EMP2 3′ UTR MT and TBX4 3′ UTR MT. Luciferase reporter vectors were transfected with either negative control or synthetic ocu-miR-29-5p mimics.

Luciferase activity was measured 48 h after transfection using the Dual-Luciferase Reporter Assay System following the manufacturer's instructions. Luciferase activity were detected using a dual-luciferase reporter assay system (Promega) and a Modulus Single Tube Multimode Reader (Turner Biosystems, Sunnyvale, CA).

### Statistical Analysis

All data were analyzed in the GraphPad Prism (version 8.0) software. The results are exhibited as the mean ± SD (standard deviation). Differences between groups were statistically analyzed using Student's *t*-tests. Thresholds for statistical significance between groups were indicated by ^*^P < 0.05; ^**^P < 0.01.

## Results

### Overview of the miRNA Sequencing Data

**Figure 1:**
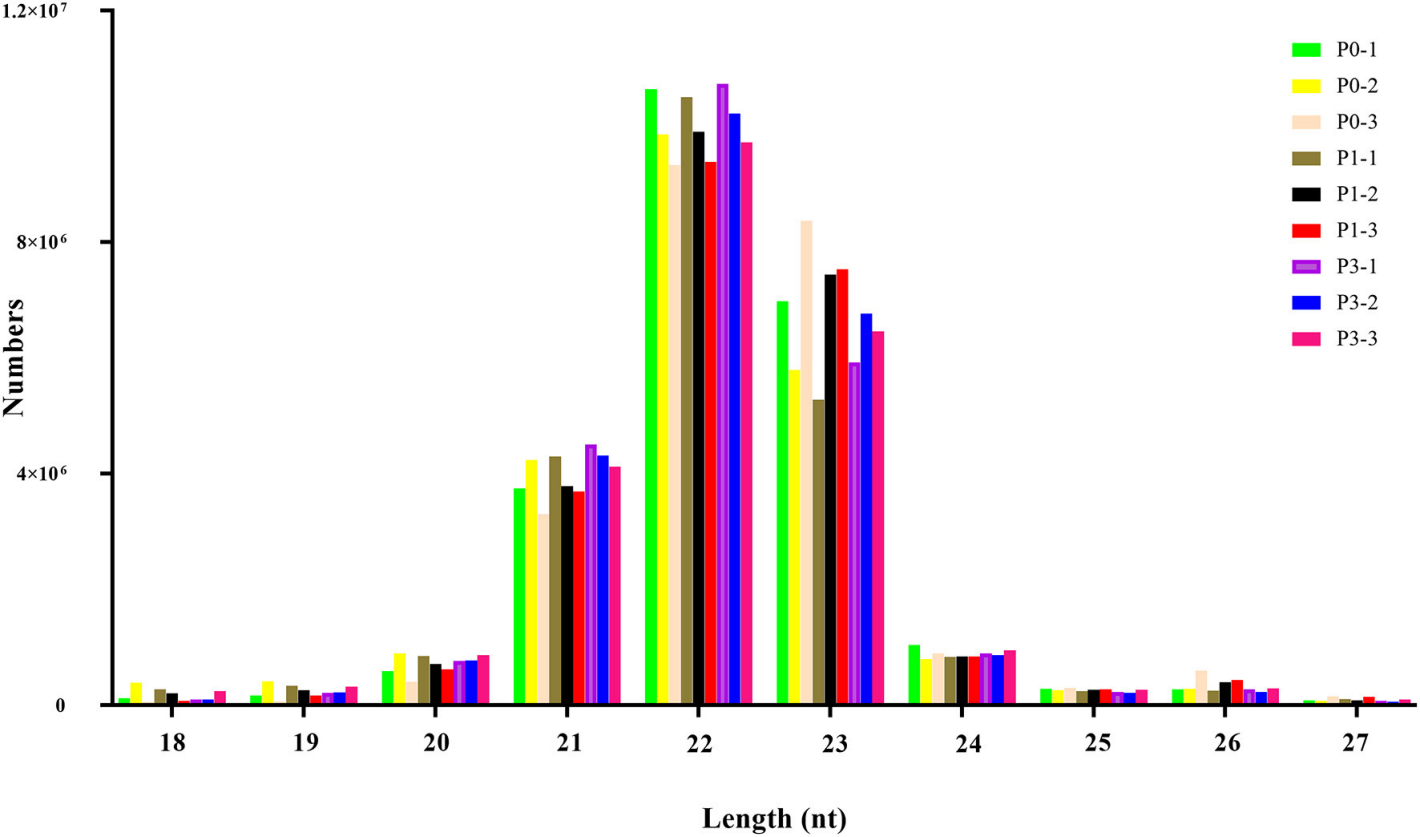
The distribution of small RNAs relative to length in the three groups of New Zealand Rabbits lung.

To obtain miRNAs related to *P. multocida* insults, we performed miRNA sequencing by the BGISEQ-500 system. Unreliable sequences were removed from raw reads, and high-quality clean reads were compared with the rabbit reference genome to map miRNA reads (Table 2). Q 30 base percentage was greater than 94%, and clean data exceeded 22.28 M for each sample. Further, the average similarity of samples to the genome was 80%. Pearson's correlation coefficient (R) across samples ranged from 0.984 to 0.997 (Supplementary Table S10), indicating reliable sequencing data for subsequent small-RNA analysis. Finally, clean reads of 18–30 nt for the three group libraries were similar in size distribution and frequency, and most sequences were 21–24 nt; sequences of 22 nt were most abundant (Figure 1). These results confirmed the homogeneity and uniformity of the sequencing data in the nine libraries. Three comparison datasets were set based on time after *P. multocida* challenge—P0-vs.-P1, P0-vs.-P3, and P3-vs.-P1. Raw reads were deposited to the NCBI database (SRA: SRP315150).

**Table 2:** Overview of miRNA sequencing data. P0, P1, and P3 in the table represent the groups 0 dpi, 1 dpi, and 3 dpi.

**Sample**	**Raw reads**	**O 30 (%)**	**Clean reads**	**Total mapping (%)**	**GC (%)**
P0-1	25165824	94.94	24087614	78.52	43.42
P0-2	25165824	95.14	23600941	80.87	44.57
P0-3	25165824	95.43	23931729	83.15	42.44
P1-1	25165824	95.75	23360526	78.61	44.16
P1-2	25165824	97.92	24173454	80.63	42.88
P1-3	25165824	95.35	23931052	81.56	42.95
P3-1	25165824	95.73	23853080	76.36	44.03
P3-2	25165824	95.08	23878350	79.34	43.88
P3-3	25165824	95.45	23645996	80.95	43.80

### sRNA Classification and miRNA Identification

To determine the miRNA sequences in the reads, miRDseep2 software package was applied. The mean miRNA reads accounted for 41.20, 40.02, and 37.78% of clean reads in the P0, P1, and P3 datasets, respectively. Additionally, unknown small RNAs made up 1.35, 1.47, and 1.23% of clean reads (Table 3), and their functions should be studies.

**Table 3:** Distribution of the small RNA among different categories.

**Sample name**	**miRNA**	**rRNA**	**snoRNA**	**tRNA**	**snRNA**	**Repeat**	**Other**	**UnmappedSmall RNA**	**Total**
P0-1	9941552	48921	21390	28543	1412	371576	6573638	282670	24087614
	41.27%	0.20%	0.09%	0.12%	0.01%	1.54%	55.59%	1.17%	100%
P0-2	9014794	134258	21281	23680	2108	1223288	12813832	367700	23600941
	38.20%	0.57%	0.09%	0.10%	0.01%	5.18%	54.3%	1.56%	100%
P0-3	10571159	109569	26325	104965	2444	594079	12204116	319072	23931729
	44.14%	0.46%	0.11%	0.44%	0.01%	2.49%	50.99%	1.33%	100%
P1-1	8609100	42566	19731	39238	2047	527759	13720456	399629	23360526
	36.85%	0.18%	0.08%	0.17%	0.01%	2.26%	58.74%	1.71%	100%
P1-2	10016502	91825	39308	52339	2048	618504	13074933	277995	24173454
	41.44%	0.38%	0.16%	0.22%	0.01%	2.56%	54.08%	1.15%	100%
P1-3	9998826	121431	31244	184939	4637	647696	12573212	369067	23931052
	41.78%	0.51%	0.13%	0.77%	0.02%	2.71%	52.54%	1.54%	100%
P3-1	9281313	34761	32293	21984	1300	287293	13910471	283665	23853080
	38.91%	0.15%	0.14%	0.09%	0.01%	1.2%	58.31%	1.19%	100%
P3-2	8972725	28804	23083	16017	1475	287042	14305992	243212	23878350
	37.58%	0.12%	0.1%	0.07%	0.01%	1.2%	59.9%	1.02%	100%
P3-3	8710611	58973	20381	25369	2896	593624	13885319	348823	23645996
	36.84%	0.25%	0.09%	0.11%	0.01%	2.51%	58.71%	1.48%	100%

**Figure 2:**
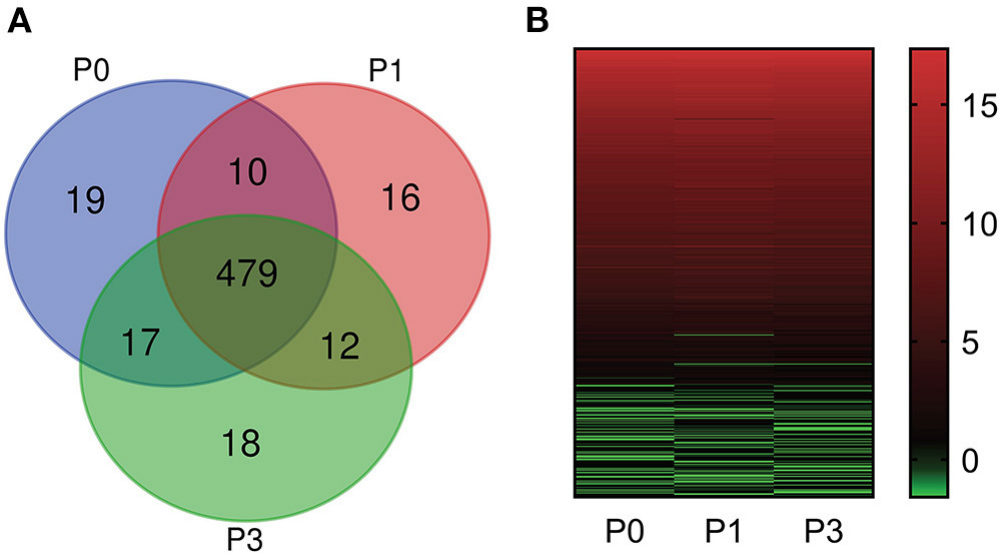
miRNA expression profiles. **(A)** Comparison of miRNA expression in lung of New Zealand Rabbits at different times post induction (Venn diagram). P0, P1, and P3 in the figure represent the groups 0 dpi, 1 dpi, and 3 dpi. **(B)** Heat map is based on the expression of miRNAs FPKM-based data from rabbit lung at 1 dpi and 3 dpi with *P. multocida*, compared to unchallenged controls (0 dpi); and drawn with GraPhpad Prism 8 software. Red indicates higher relative expression and green indicates lower relative expression.

Bioinformatic analysis of sequencing data across all samples yielded 571 miRNAs, consisting of 509 known and 62 new miRNAs (Supplementary Table S1). Notably, expression of DE-miRNA at 0, 1, and 3 dpi following *P. multocida* challenge showed 19 (10 known and 9 novel), 16 (12 known and 4 novel), and 18 (9 known and 9 novel) specifically expressed in lung tissues (Figure 2A), respectively. These miRNAs may have critical functions for modulating responses to infection at different stages of infection.

### miRNA Expression Overall Distribution

We determined FPKM values at three times based on deep sequencing data. miRNA expression profiles are illustrated in Figure 2B. The miRNA expression changed over time, demonstrating that DE-miRNAs play distinct roles from onset to progression of *P. multocida* infection. Further, the top six highly expressed miRNAs were identified (FPKM > 100000) (Supplementary Table S2): ocu-miR-451-5p, ocu-miR-26a-5p, ocu-miR-143-3p, ocu-let-7a-5p, ocu-miR-22-3p, and ocu-let-7f-5p.

### DE-miRNA Analysis

**Figure 3:**
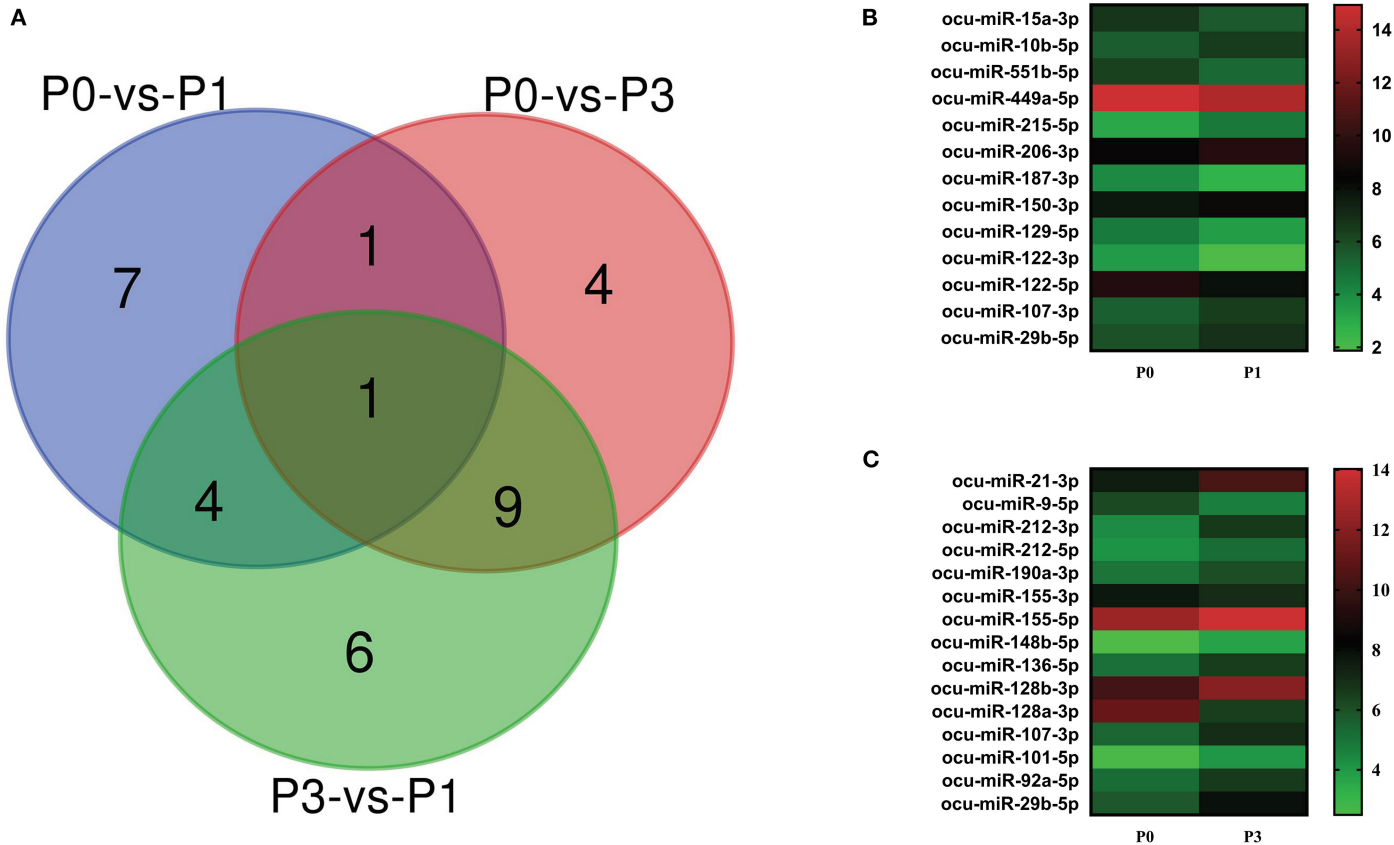
**(A)** Venn diagram showing the DE miRNAs in the three comparison groups: P0-vs-P1, P0-vs-P3, and P3-vs-P1. Heat map analysis of DE-miRNAs. Based on the sequenced FPKM data from rabbit lung at 1 dpi **(B)** and 3 dpi **(C)** with *P. multocida*, compared to unchallenged controls (0 dpi). Heat map was drawn using GraPhpad Prism 8 software. Red indicates higher relative expression and green indicates lower relative expression. From bottom to top, the |log2(FC)| gradually increased.

We carried out paired comparative analysis based on the three FPKM datasets, using |log2(Fold Changes)| ≥ 1, Q value ≤ 0.001, and FPKM > 1 as strict selection criteria, and identified 32 DE-miRNAs (Figure 3A, Supplementary Table S3). All DE-miRNAs were previously recognized sequences. Pairwise comparisons among P0, P1, and P3 datasets indicated 13 miRNAs (6 upregulated and 7 downregulated) for the P0-vs.-P1, 15 (13 upregulated and 2 downregulated) for the P0-vs.-P3, and 20 (6 upregulated and 14 downregulated) for the P3-vs.-P1. A Venn diagram was created to depict overlaps among these comparisons. Seven, four, and six time-specific DE-miRNAs were noted, respectively.

Notably, the expression of ocu-miR-107-3p (log2 ^FC^ = 1.02 and 1.60) and ocu-miR-29b-5p (log2 ^FC^ = 1.07 and 2.13) in P1 and P3 datasets were significantly increased compared with P0. At P1, miRNA upregulated to the greatest extent was ocu-miR-215-5p (log2 ^FC^ = 1.47), and miRNA ocu-miR-122-3p (log2 ^FC^ = −1.71) was the most downregulated. However, miRNAs ocu-miR-21-3p (log2 ^FC^ = 2.83) and ocu-miR-128a-3p (log2 ^FC^ = −4.66) replaced these miRNAs, respectively, at P3 (Figures 3B,C). These miRNAs may be involved in the complex regulation of responses to *P. multocida* infection.

### DE-miRNAs Target Genes Prediction and GO and KEGG Analysis

**Figure 4:**
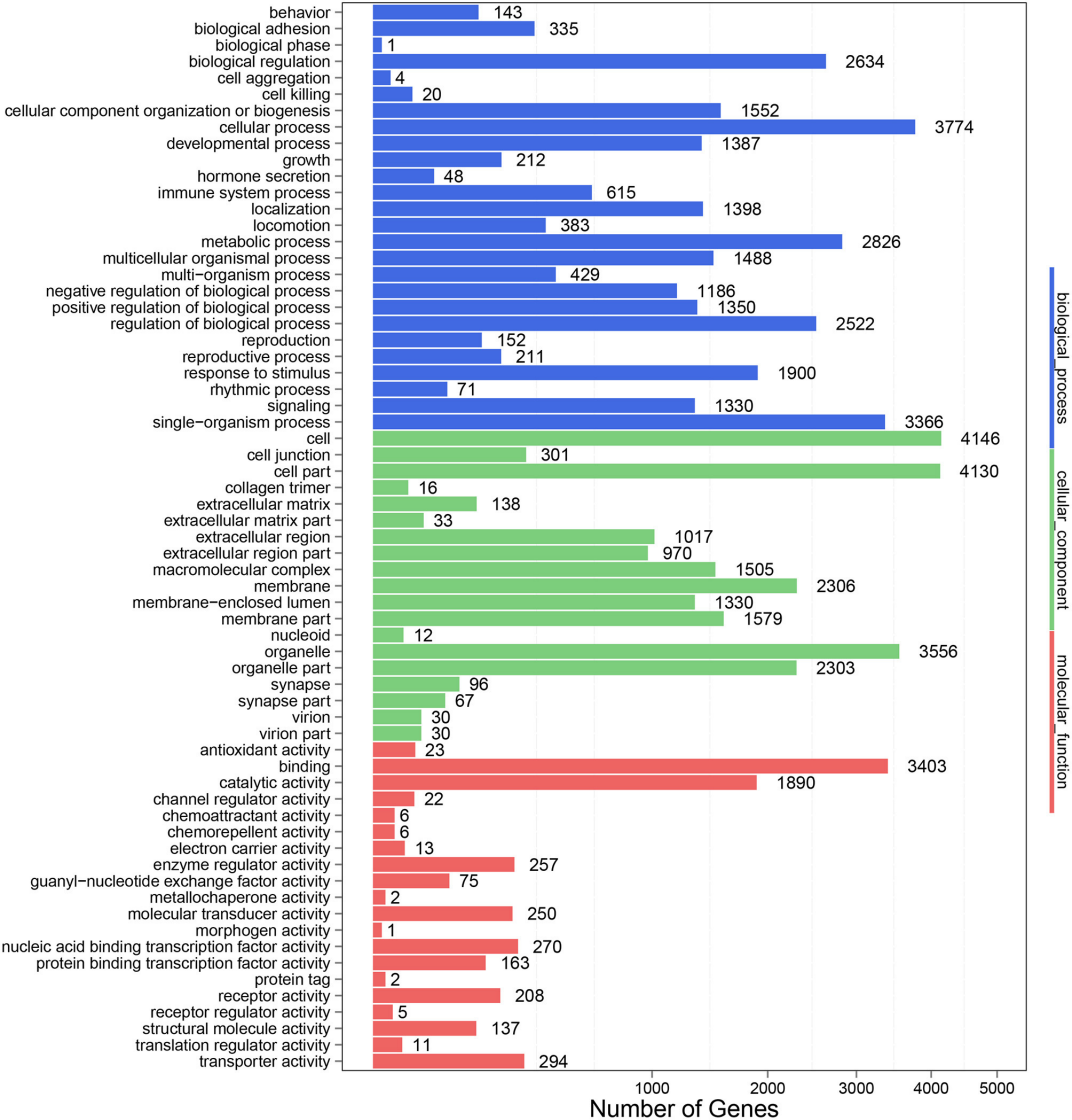
GO functional classification of DE-miRNA target genes in response to *P. multocida* insults. The number of DEGs is shown on the X axis, while GO terms are shown on the Y axis. All GO terms are grouped into three ontologies: biological processes are shown in blue, cellular components in green, and molecular functions in red.

**Figure 5:**
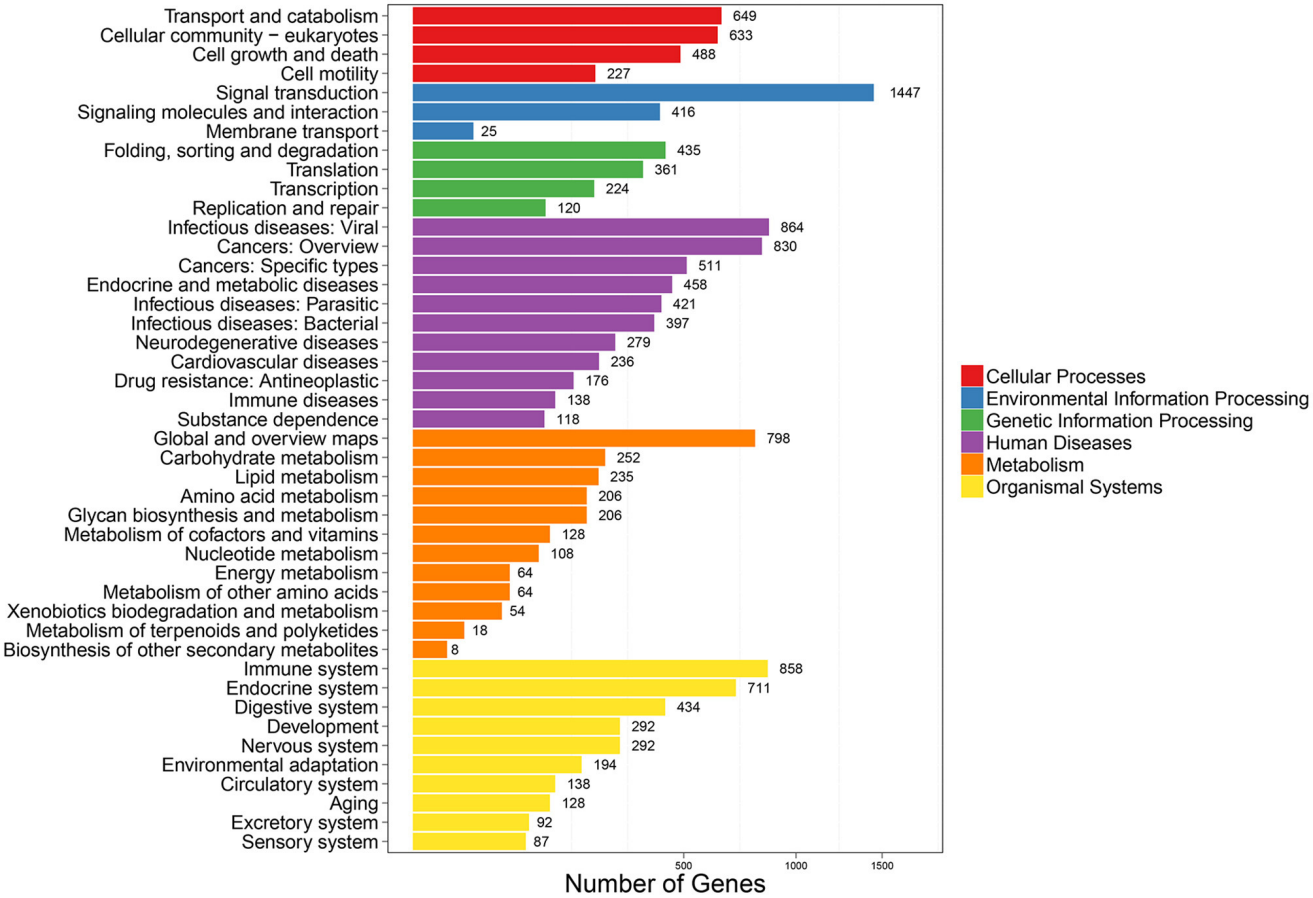
KEGG pathway enrichment analysis of DE-miRNA target genes. The number of DEGs are shown on the X axis, while the KEGG pathway terms are shown on the Y axis. All second pathway terms are grouped in the following top pathway terms, as indicated by different colors.

**Figure 6:**
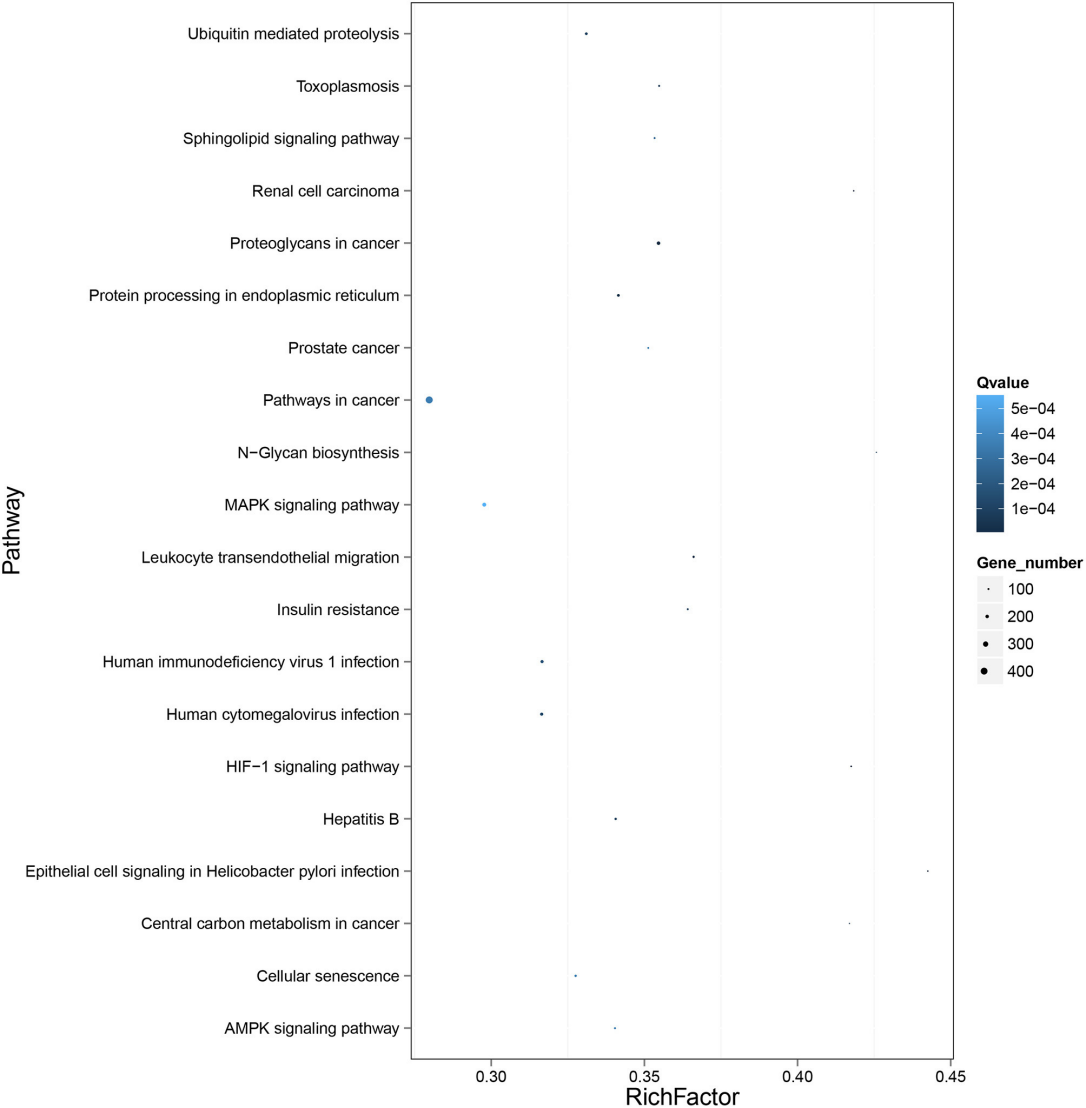
KEGG pathway enrichment analysis of the DE mRNAs with the 20 highest rich ratios. The size of dots indicates the number of enriched genes, and the color of dots indicates the degree of enrichment. Higher rich ratio correlate with lower Q-values, indicating that the enrichment of DE genes in a given pathway is significant.

To further clarify the role of DE-miRNAs, we predicted the DE-miRNA's target genes. The 32 DE-miRNAs may have 7,345 non-redundant targets (Supplementary Table S4). DE-miRNAs' target genes are associated with 65 GO terms (26 biological process, 19 cellular component and 20 molecular function) (Figure 4, Supplementary Table S5). The primary enriched biological process, cellular component, and molecular function GO terms were macromolecule metabolic process, intracellular, and binding, respectively. Moreover, a remarkable 130 KEGG pathways were associated with target genes of DE-miRNAs (Figure 5, Supplementary Table S6). Ubiquitin-mediated proteolysis, MAPK signaling, and proteoglycans in cancer were the most notable KEGG pathways (Figure 6). miRNAs have a range of functions and may participate in *P. multocida* defense by targeting a variety of pathways and genes.

### DE-miRNA RT-qPCR Validation

**Figure 7:**
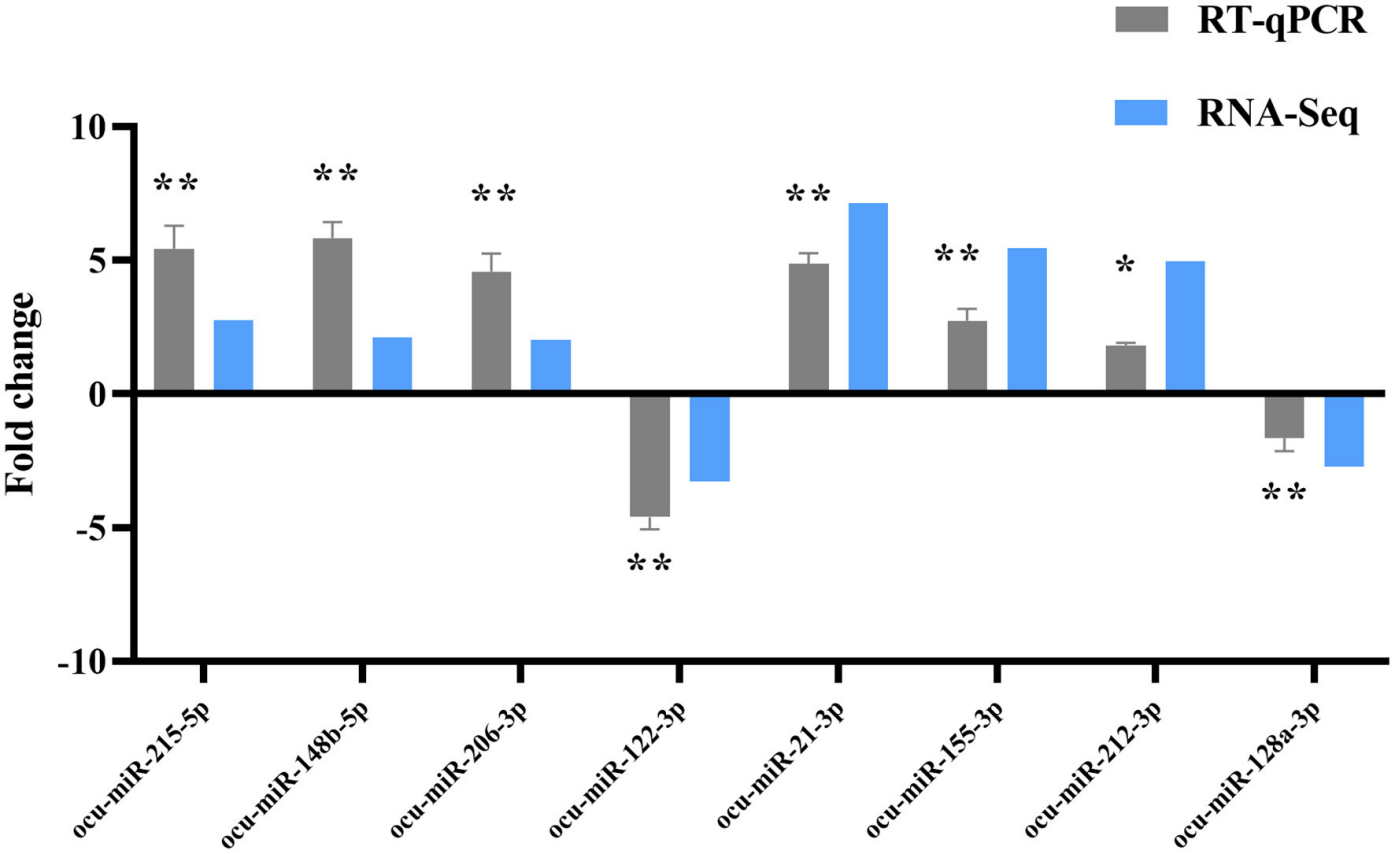
RT-qPCR validation of DE-miRNAs. Compared with the P0, *P < 0.05, **P < 0.01.

Eight DE-miRNAs were randomly selected, and RT-qPCR data were compared with the deep high-throughput sequencing data (Figure 7). Concordant results in RNA-seq and RT-qPCR analysis confirmed the reliability of sequencing for identifying DE-miRNAs.

### Expression Profile of miR-29-5p in Different Tissues

Among the DE-miRNAs, ocu-miR-29b-5p was upregulated at both time points and was selected for intensive exploration for a more comprehensive understanding of predicted targets. EMP2 and TBX4 are targets of ocu-miR-29b-5p, and the expression and function were further clarified. Next, changes in the expression of ocu-miR-29b-5p in infected rabbit tissues, including the lung, spleen, liver, and kidney, were quantified.

**Figure 8:**
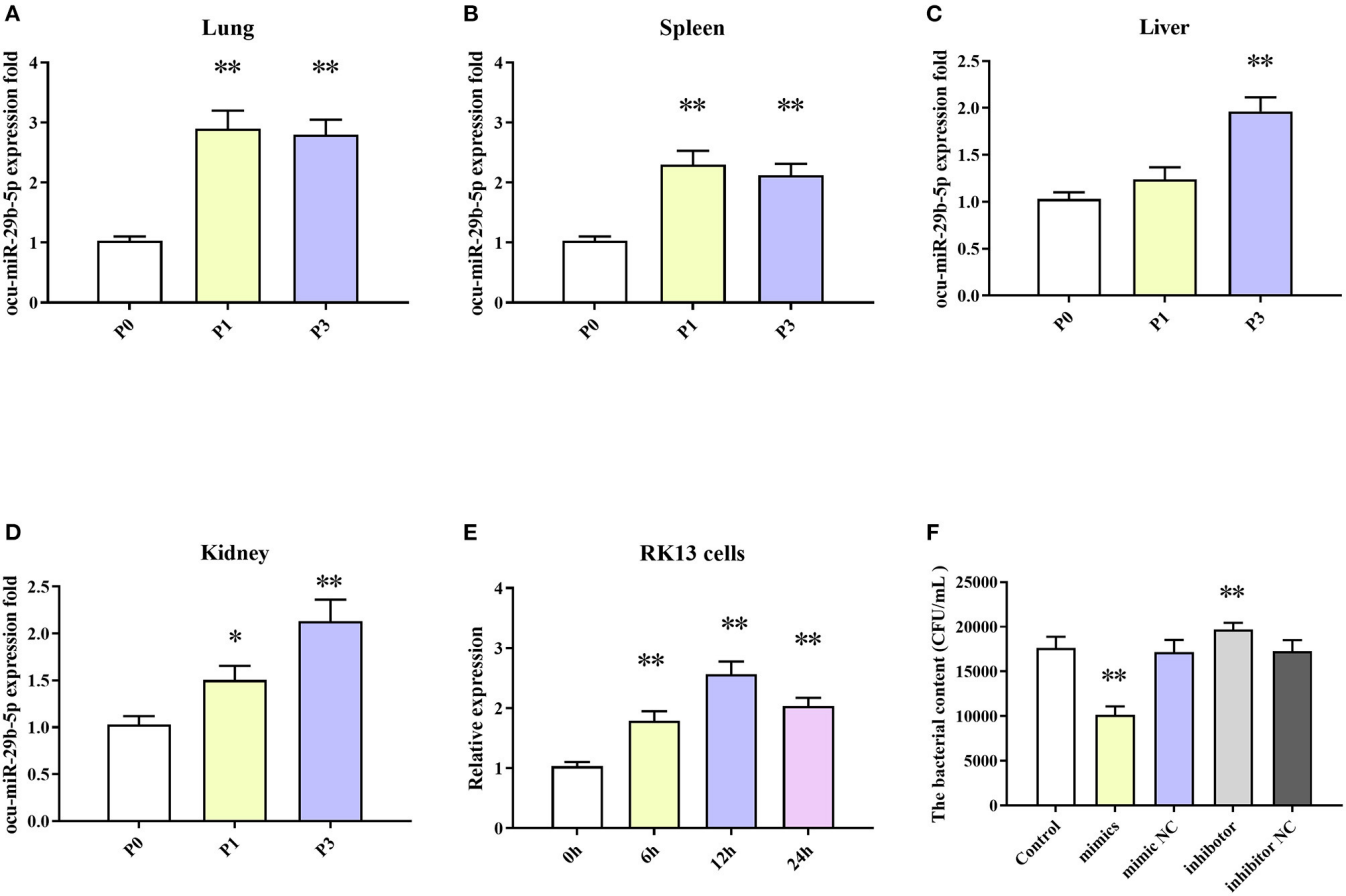
ocu-miR-29b-5p expression profiles and effect of ocu-miR-29b-5p on the infectivity of *P. multocida*. The time-coures expression profiles of ocu-miR-29b-5p in the lung **(A)**, spleen **(B)**, liver **(C)**, kidney **(D)**, and RK13 cells **(E)**. RK13 cells were treated with *P. multocida* in the presence of ocu-miR-29b-5p mimics or knockdown of miR-29b-5p, and bacterial recovery from the cells was counted by plate count **(F)**. Values are shown as means ± SD. Compared with the P0, *P < 0.05, **P < 0.01.

The results indicated that expression levels of ocu-miR-29b-5p in all four tissues were markedly elevated after *P. multocida* challenge. Specifically, ocu-miR-29b-5p was rapidly upregulated and reached a peak at 1 dpi in the lung and spleen samples (Figures 8A,B). Expression of ocu-miR-29b-5p in the liver was significantly increased and reached a peak at 3 dpi (Figure 8C). Consistently, expression of ocu-miR-29b-5p in kidney samples was also a time-dependent upregulation (Figure 8D). Moreover, *P. multocida* challenge stimulated RK13 cells and induced ocu-miR-29b-5p expression, which was significantly upregulated over time, with ocu-miR-29b-5p expression reaching a peak value at 12 h post-infection (Figure 8E).

### MiR-29-5p Can Repress *P. multocida* Proliferation

Due to *P. multocida* induced enhanced ocu-miR-29b-5p expression, we wondered whether ocu-miR-29b-5p would affect the infectivity of *P. multocida*. To explicit this doubt, RK13 cells transfected with or without synthetic ocu-miR-29b-5p mimic and inhibitor were challenged with *P. multocida*. Subsequent *P. multocida* invasion analysis indicated that bacterial loads in ocu-miR-29b-5p mimic-transfected cells were significantly lower than in control cells, while inhibited expression of ocu-miR-29b-5p promoted the bacterial loads in PK13 cells. Hence, ocu-miR-29b-5p does reduce *P. multocida* propagation in RK13 cells (Figure 8F).

### EMP2 and TBX4 Were Directly Targeted by miR-29-5p

**Figure 9:**
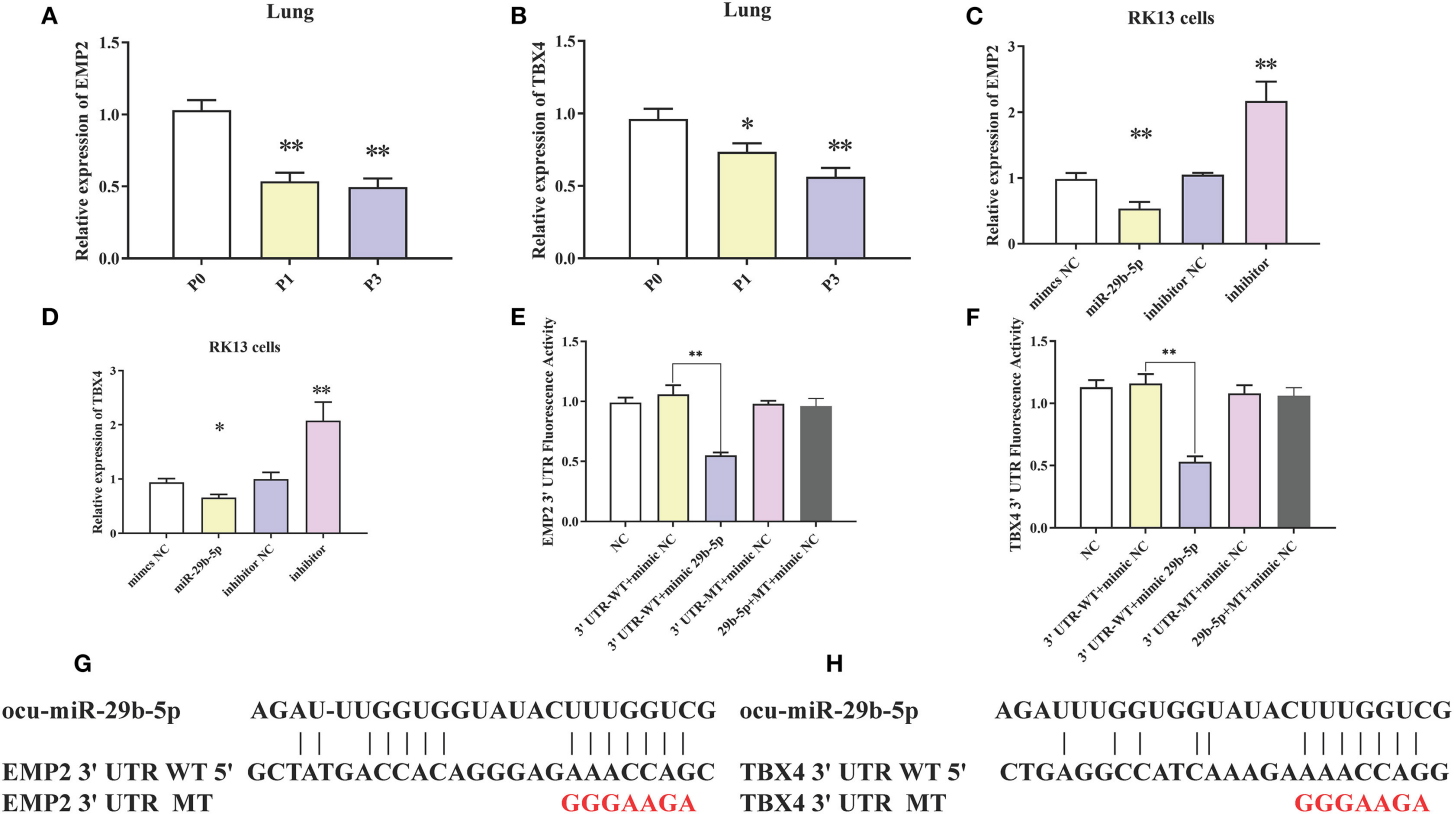
Confirmation of EMP2 and TBX4 are direct targets of ocu-miR-29b-5p. EMP2 **(A)** and TBX4 **(B)** expression profiles in rabbit infected or uninfected lung samples. EMP2 **(C)** and TBX4 **(D)** expression profilers in RK13 cells transfected with ocu-miR-29b-5p mimics or knockdown of miR-29b-5p. Relative fluorescence activity of the luciferase constructs containing the wildtype or mutant 3′ UTR of EMP2 **(E)** and TBX4 **(F)** genes in RK13 cells with or without the ocu-miR-29b-5p mimic. Diagram of the potential ocu-miR-29b-5p binding site within the 3′ UTR of EMP2 **(G)** and TBX4 **(H)** (3′ UTR WT). The mutated 3′ UTR (3′ UTR MT) harbors a mutated region that could not complementary to the ocu-miR-29b-5p seed sequence (GGGAAGA). Values are shown as means ± SD. *P < 0.05, **P < 0.01.

To clarify the regulatory relationship between ocu-miR-29b-5p and the targets, expression levels of EMP2 and TBX4 in the rabbit lung samples were quantified. EMP2 was markedly decreased in *P. multocida*-infected rabbit lung, opposite to the expression of ocu-miR-29b-5p (Figure 9A). Similar changes were observed for the relative expression of TBX4 (Figure 9B). Furthermore, the transfection of ocu-miR-29b-5p significantly reduced expression levels of EMP2 and TBX4, whereas knockdown expression of ocu-miR-29b-5p significantly promoted the expression of EMP2 and TBX4 (Figures 9C,D). Following, we confirmed that EMP2 and TBX4 are direct targets of ocu-miR-29b-5p indicated by the vector psiCheck2 linked to wild or mutant 3′ UTRs of rabbit EMP2 and TBX4 genes, respectively. Co-transfection of luciferase constructs with the ocu-miR-29b-5p mimic showed that the ocu-miR-29b-5p mimic markedly reduced luciferase activity from constructs harboring the wild type 3′ UTR of both EMP2 and TBX4, but not the mutant 3′ UTR of these genes (Figures 9E,F). Collectively, ocu-miR-29b-5p negatively regulates the expression of the EMP2 and TBX4 genes through directly specific binding to their 3′ UTR (Figures 9G,H).

## Discussion

*P. multocida* challenge induces severe respiratory illness that diminishes productivity and restricts rabbit industry prosperity (5, 36, 37). Hence, understanding the mechanisms of action of *P. multocida* infection at the molecular level is an urgent need. miRNA is involved in many biological functions, ranging from immune responses to infection, stress responses, and development (15, 38). Numerous reports have documented miRNAs as pivotal effectors for crosstalk between pathogenic bacteria and their hosts (16, 17, 19). To explicit crucial miRNAs participated in the *P. multocida* attacking, miRNA expression profiles in infected rabbit lungs tissues were generated.

Length distribution is useful in identifying miRNAs. The typical length of miRNAs ranges from 18 to 30 nt, with animal miRNAs displaying 22 nt. miRNA from 21 to 24 nt were responsible for 88%−92% of all reads in each library (Figure 1), reflecting miRNA plurality. Moreover, predominant miRNAs in libraries were 22-nt long, consistent with earlier findings from other research with rabbit (39), pig (40) and cow (41). Thus, nine miRNA libraries developed from rabbit tissues were technically reliable and suitable for subsequent investigation.

*P. multocida* challenge stimulates miRNAs expression. Totally, 571 miRNAs were detected, consisting of 509 known and 62 newly predicted (Figures 2A,B, Supplementary Table S1). Comparison of miRNA expression revealed that 32 miRNAs were DE, including 6 upregulated and 7 downregulated in the P0-vs-P1 group, 13 upregulated and 2 downregulated in the P0-vs-P3 group (Figures 3A–C, Supplementary Table S2). The identified both frequent and unique miRNAs showed that expression of miRNA is complex when *P. multocida* is encountered. Interestingly, ocu-miR-107-3p and ocu-miR-29b-5p are simultaneously upregulated at two time points, suggesting involvement in crosstalk with host. Previous investigations indicate that miR-107 is indispensable for diverse biological processes, e.g., cell differentiation (42), response to chemotherapy (43), insulin resistance (44), and metastasis (45, 46). Growing evidence implicates elevated miR-107 as a prognostic risk factor for multiple malignant diseases, including gastric cancer (47), oropharyngeal cancer (48), colorectal cancer (49), and breast cancer (50). Moreover, miR-29b, an miR-29 family member, promotes the appearance and progression of many diseases. For example, miR-29b positively modulates adipogenesis resulting in obesity (51), and miR-29b repression mitigates the progression of abdominal aortic aneurysm (52). In addition, miR-29b also regulates cell–cell adherence, promoting migration of oral squamous cell carcinoma cells and downregulating CX3CL1 (53). Thus, we speculate that it could be a valuable target for antibacterial treatment or prophylaxis.

miRNA manipulate gene expression by binding to the 3′-UTR of target genes (11). Whereas, most miRNAs exert their regulatory roles not only via a single gene, but a huge network consisting of various genes (32).

Thus, exploring miRNA–mRNA modulatory networks using GO terms and KEGG pathways might help identify biological roles linked to immune responses to *P. multocida* challenge. GO analysis illustrated that target genes of miRNAs were particularly linked to the macromolecule metabolic process, binding, and intracellular (Figure 4), all involved in immune responses. Further, ubiquitin-mediated proteolysis, MAPK signaling, and proteoglycans in cancer were the top three enriched KEGG pathways (Figures 5, 6). Hence, linking specific expression of crucial miRNA target genes to survey the biology of infection will provide substantial insight into the response to *P. multocida* insult.

Our miRNA-sequence findings show that miRNA-29b-5p upregulation is shared between time points after *P. multocida* challenge. Accordingly, we speculate that activated miRNA-29b-5p participates in rabbit defenses against *P. multocida* invasion.

However, no relevant information is available for the function of miRNA-29b-5p in regulating immune response, especially combating with *P. multocida* infection in rabbits. Therefore, novel findings will provide significant information for the core mechanisms of action in regulating immune responses in these animals. miRNA is known to regulate gene expression by targeting 3′-UTR (11). Consequently, the identification of miRNA targets is essential for clarification of the regulatory roles of individual miRNA.

Initially, we measured miRNA-29b-5p expression levels in multiple organs (lung, spleen, liver, and kidney) and RK13 cells to demonstrate the functional roles of miRNA-29b-5p. This miRNA was elevated in several tissues and in RK13 cells during *P. multocida* infection (Figures 8A–E). Interestingly, when miRNA-29b-5p is overexpressed in RK13 cells, *P. multocida* propagation is significantly inhibited. While knockdown of miR-29b-5p, *P. multocida* propagation is promoted (Figure 8F). Moreover, examination of likely targets of miRNA-29b-5p confirmed that EMP2 and TBX4 expression were negatively regulated by miRNA-29b-5p through direct binding with 3′ UTR sequences (Figures 9A–F).

EMP2, a novel tumor-related protein, encodes an interesting integral tetraspan membrane protein that is vital for cancer progression (54), innate immune response (55) and neutrophil transmigration (56). EMP2 is aberrantly upregulated in multiple human cancers, including 63% of invasive breast cancers (57). EMP2 also suppresses non-small cell lung cancer cell growth by inhibition of MAPK pathway (58). Additionally, EMP2 modulates innate immune cell population recruitment at the maternal-fetal interface (55). In the present study, EMP2 expression was reduced due to miRNA-29b-5p expression in rabbit lung tissue. During bacterial pneumonia in mice, knockout of EMP2 attenuated neutrophilic lung injury and improved survival (56). TBX4 belongs to the ancient T-box family of transcription factors that drives the accumulation of myofibroblasts and the development of pulmonary fibrosis (59). TBX4 is ubiquitously expressed in several tissues and is crucial for organogenesis, especially in proper lung organogenesis (60). Moreover, TBX4 play a key role in systemic lupus erythematosus (SLE) disease pathogenesis through mediating abnormal T cell activity (61). In mouse models, specific knockout of TBX4 or perturbation of TBX4 gene function in fibroblasts markedly relieved lung fibrosis after bleomycin-induced damage (59). Additional exploration is needed to clarify whether EMP2 and TBX4 directly control responses to *P. multocida* infection.

## Conclusion

In summary, we first report a comprehensive assessment of rabbit miRNA expression in the course of *P. multocida* infection. Findings are reliable and mimic biological processes in naturally infected rabbits; the host immune system is a vital component of host-*P. multocida* crosstalk. Further, 32 DE-miRNAs are associated with diverse functions, in particular, immunity, and one crucial miRNA was involved in immune defense against *P. multocida*. In RK13 cells, miRNA-29b-5p represses *P. multocida* propagation, and miRNA-29b-5p may downregulate the expression of EMP2 and TBX4 by binding to their 3′ UTR. These observations add to the current understanding of the roles and mechanisms of miRNAs in *P. multocida*-induced infection in rabbits and humans.

## Data Availability Statement

The datasets presented in this study can be found in online repositories. The names of the repository/repositories and accession number(s) can be found in the article/Supplementary Material.

## Ethics Statement

All animal studies were conducted according to the guidelines of the Institutional Animal Care and Use Committee of Shandong Agricultural University and the Guidelines for Experimental Animals of the Ministry of Science and Technology (Beijing, China).

## Author Contributions

JH, XF, BH, and QZ designed this research. JH, XQ, WQL, WJL, YW, and KX took the samples, conducted the experiments, and write the manuscript. JH and XF modified the manuscript. LL responded to the comments and conducted additional verification experiments raised by reviewers and polished the language of the manuscript. All authors contributed to the article and approved the submitted version.

## Funding

This study was supported by the Shandong Province Special Economic Animal Innovation Team (Grant Numbers SDAIT-21-02 and SDAIT-21-16), Natural Science Foundation of Shandong Province (Item no. ZR202102200454), High-level Scientific Research Foundation for the introduction of talent of Shandong Agricultural University (Grant Number: 72237), and Funds of Shandong Double Tops Program (Grant Number SYL2017YSTD12).

## Conflict of Interest

XQ was employed by the company Shandong New Hexin Technology Co. Ltd. The remaining authors declare that the research was conducted in the absence of any commercial or financial relationships that could be construed as a potential conflict of interest.

## Publisher's Note

All claims expressed in this article are solely those of the authors and do not necessarily represent those of their affiliated organizations, or those of the publisher, the editors and the reviewers. Any product that may be evaluated in this article, or claim that may be made by its manufacturer, is not guaranteed or endorsed by the publisher.
